# Case Report: Tachycardia, Hypoxemia and Shock in a Severely Burned Pediatric Patient

**DOI:** 10.3389/fcvm.2022.904400

**Published:** 2022-06-16

**Authors:** Jianshe Shi, Chuheng Huang, Jialong Zheng, Yeqing Ai, Hiufang Liu, Zhiqiang Pan, Jiahai Chen, Runze Shang, Xinya Zhang, Shaoliang Dong, Rongkai Lin, Shurun Huang, Jianlong Huang, Chenghua Zhang

**Affiliations:** ^1^Department of Surgical Intensive Care Unit, Huaqiao University Affiliated Strait Hospital, Quanzhou, China; ^2^Department of General Surgery, Huaqiao University Affiliated Strait Hospital, Quanzhou, China; ^3^School of Medicine, Huaqiao University, Quanzhou, China; ^4^Department of Burn, Huaqiao University Affiliated Strait Hospital, Quanzhou, China; ^5^Key Laboratory of Intelligent Computing and Information Processing, Quanzhou Normal University, Quanzhou, China

**Keywords:** tachycardia, hypoxemia, shock, abdominal compartment syndrome, pediatric, severe burns, continuous renal replacement therapy

## Abstract

**Background:**

Severely burned children are at high risk of secondary intraabdominal hypertension and abdominal compartment syndrome (ACS). ACS is a life-threatening condition with high mortality and requires an effective, minimally invasive treatment to improve the prognosis when the condition is refractory to conventional therapy.

**Case presentation:**

A 4.5-year-old girl was admitted to our hospital 30 h after a severe burn injury. Her symptoms of burn shock were relieved after fluid resuscitation. However, her bloating was aggravated, and ACS developed on Day 5, manifesting as tachycardia, hypoxemia, shock, and oliguria. Invasive mechanical ventilation, vasopressors, and percutaneous catheter drainage were applied in addition to medical treatments (such as gastrointestinal decompression, diuresis, sedation, and neuromuscular blockade). These treatments did not improve the patient's condition until she received continuous renal replacement therapy. Subsequently, her vital signs and laboratory data improved, which were accompanied by decreased intra-abdominal pressure, and she was discharged after nutrition support, antibiotic therapy, and skin grafting.

**Conclusion:**

ACS can occur in severely burned children, leading to rapid deterioration of cardiopulmonary function. Patients who fail to respond to conventional medical management should be considered for continuous renal replacement therapy.

## Case Presentation

A 4.5-year-old girl was transferred to the emergency department of our tertiary care center, with burns covering 40% of her body surface area from boiling water. She received no intravenous fluid administration within 30 h post-scalding and complained of tachycardia, dizziness, weakness, and oliguria. Her physical examination at admission showed that her blood pressure was 98/73 mmHg, her body temperature was 37.3°C, her pulse rate was 164 beats per minute, and her respiration rate was 25 breaths per minute. The pulse oxygen oximeter read 95% on room air. The patient presented with clammy extremities and an increased capillary refill time. While receiving appropriate first aid and wound assessment, she was resuscitated immediately using lactated ringer's injection based on a potential diagnosis of burn shock from her focused history taking, signs, and symptoms. Then, the patient was quicikly and gently transferred to the burn center of our hospital for further treatment.

**Figure 1:**
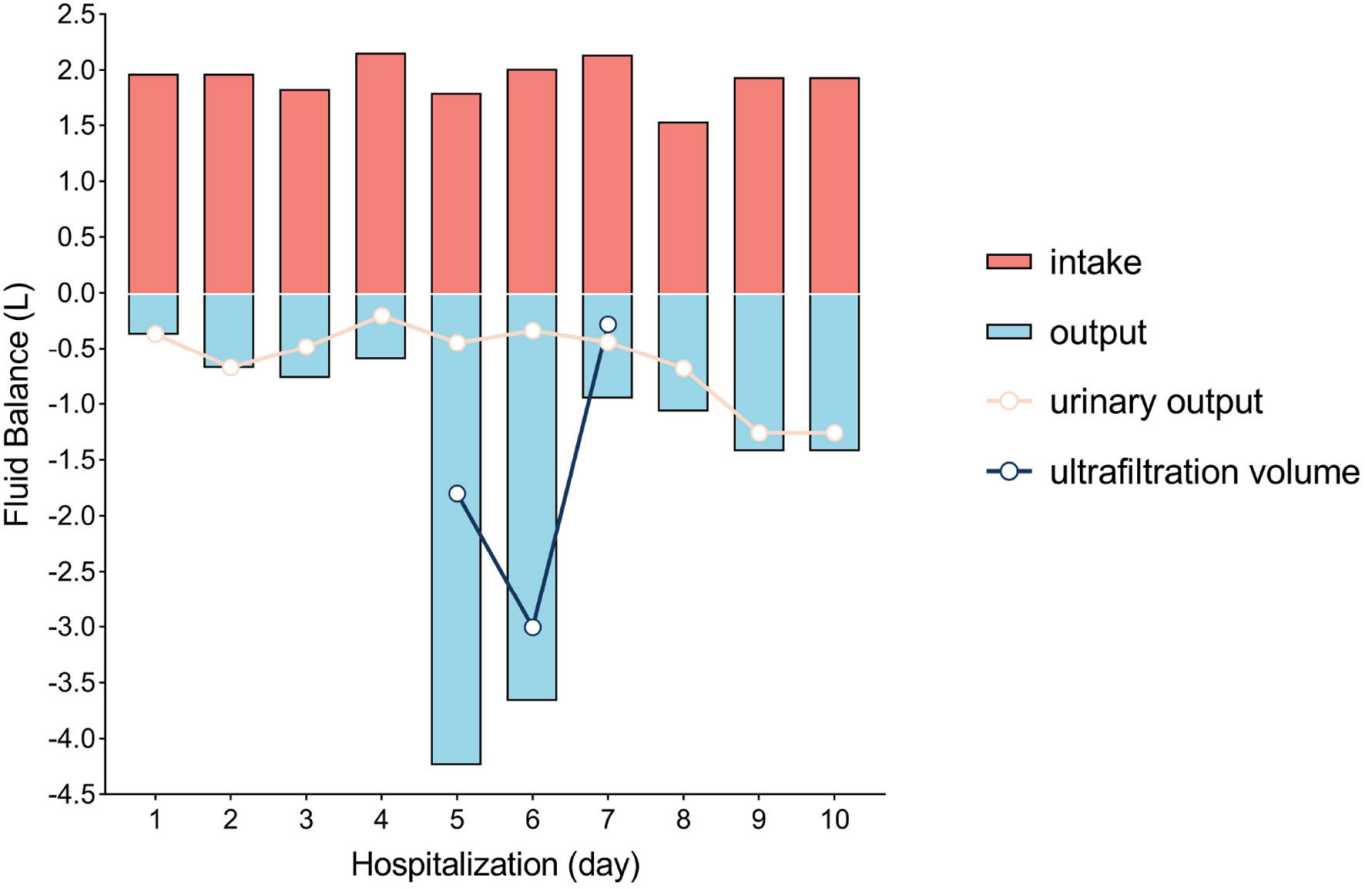
Fluid balance in the first 10 days of hospitalization. Day 1 was defined as the time between hospital admission and the next morning (14 h).

**Figure 2:**
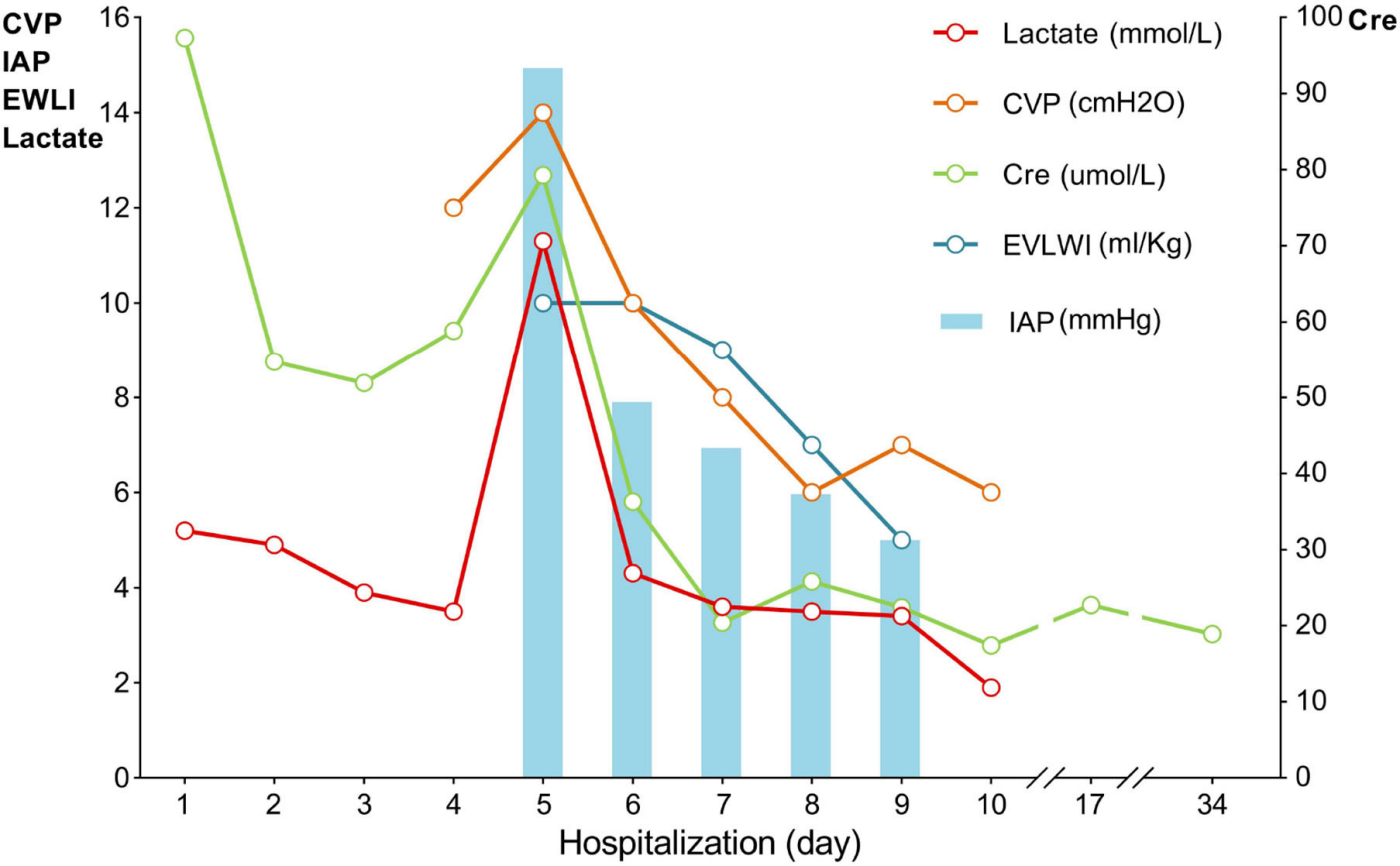
Trends of vital parameters during hospitalization. CVP, central venous pressure; IAP, intra-abdominal pressure; EVLWI, extravascular lung water index; Lac, lactate, Cre, serum creatinine.

Laboratory investigations revealed a serum creatinine level of 97.3 μmol/L and an arterial blood serum lactate level of 5.2 mmol/L. These data indicated acute kidney injury induced by hypovolemic shock and confirmed the fluid resuscitation requirement. After resuscitation to correct dangerous deficits in accordance with the Parkland Formula, the patient's vital signs, mental status, capillary refill time, and serum creatinine level improved, and reached a target of 0.5–1.0 ml/kg/h^−1^ of urine output, indicating adequate fluid resuscitation. Then, the fluid rates were adjusted accordingly based on the monitoring results. The patient's condition improved as expected within the first 2 days. When the patient showed signs of bloating on the 3rd day, a nasogastric tube was inserted, and gastrointestinal prokinetic agents were administered to prevent abdominal over distension. During the aggravation of bloating on the 4th day, the volume of fluid administration was increased in accordance with the trend of decreased urine output. The daily fluid balances are summarized in Figure 1. On the 5th day of hospitalization, she developed hypoxemia, tachypnoea, hypotension, and oliguria. Blood pressure was 75/53 mmHg, body temperature was 37.2°C, pulse rate was 155 beats per minute, and respiration rate was 45 breaths per minute. The PaO_2_/FiO_2_ ratio was 126 mmHg, with 10 L/min oxygen flow delivered by nasal cannula. Her intra-abdominal pressure (IAP) increased to and remained above 15 mmHg as measured from the intrabladder pressure. The central venous pressure increased to a level above 14 cmH_2_O, the extravascular lung water index increased to >10 ml/kg, and the B-type natriuretic peptide level was >35,000 pg/ml. The urine output was <0.3 ml/kg·h^−1^, with serum creatinine at 81 μmol/L. The patient was unresponsive to furosemide. These findings indicated the development of secondary abdominal compartment syndrome (ACS), coupled with refractory fluid overload. The patient was intubated and mechanically ventilated immediately, and norepinephrine (1.6 μg/kg·min^−1^) was administered to maintain mean arterial pressure above 70 mmHg. A neuromuscular blocking agent (cisatracurium besylate) was administered upon sedation and analgesia to improve thoracic and abdominal wall compliance. To manage the increased IAP, a percutaneous catheter was inserted into the abdominal cavity for drainage with ultrasound guidance, and 1,100 ml of fluid was drained within 28 h. The IAP decreased to 11 mmHg, but the clinical condition did not improve. Then, continuous renal replacement therapy (CRRT) was performed with an ultrafiltration flow rate of 20–25 ml/kg·h^−1^. After 40 h of hemofiltration, 5,080 ml of fluid was removed in total. IAP declined to 7 mmHg immediately and then dropped to 5 mmHg. The patient's vital signs subsequently stabilized, the B-type natriuretic peptide level decreased to 5,204 pg/ml, and urine output increased to 1.3 ml/kg·h^−1^. CRRT was terminated on Day 7, and mechanical ventilation was weaned on Day 8. The trends of the laboratory tests and vital parameters are presented in Figure 2. After recovering from ACS, the patient continued to improve under routine enteral nutrition support and antibiotic therapy. No significant infections were observed. Skin grafting was performed on Day 17. The patient fully recovered and was discharged from the hospital 34 days after her admission.

## Discussion

ACS in children is defined as a sustained elevated IAP (>10 mmHg) associated with new-onset or worsening organ dysfunction (1). Secondary ACS occurs in the absence of injury or disease in the abdominal or pelvic area. A study showed that 10–30% of patients with a burn injury, covering more than 20% of the total body surface, develop secondary ACS, and the mortality ranges from 40 to 100% (2–6).

**Figure 3:**
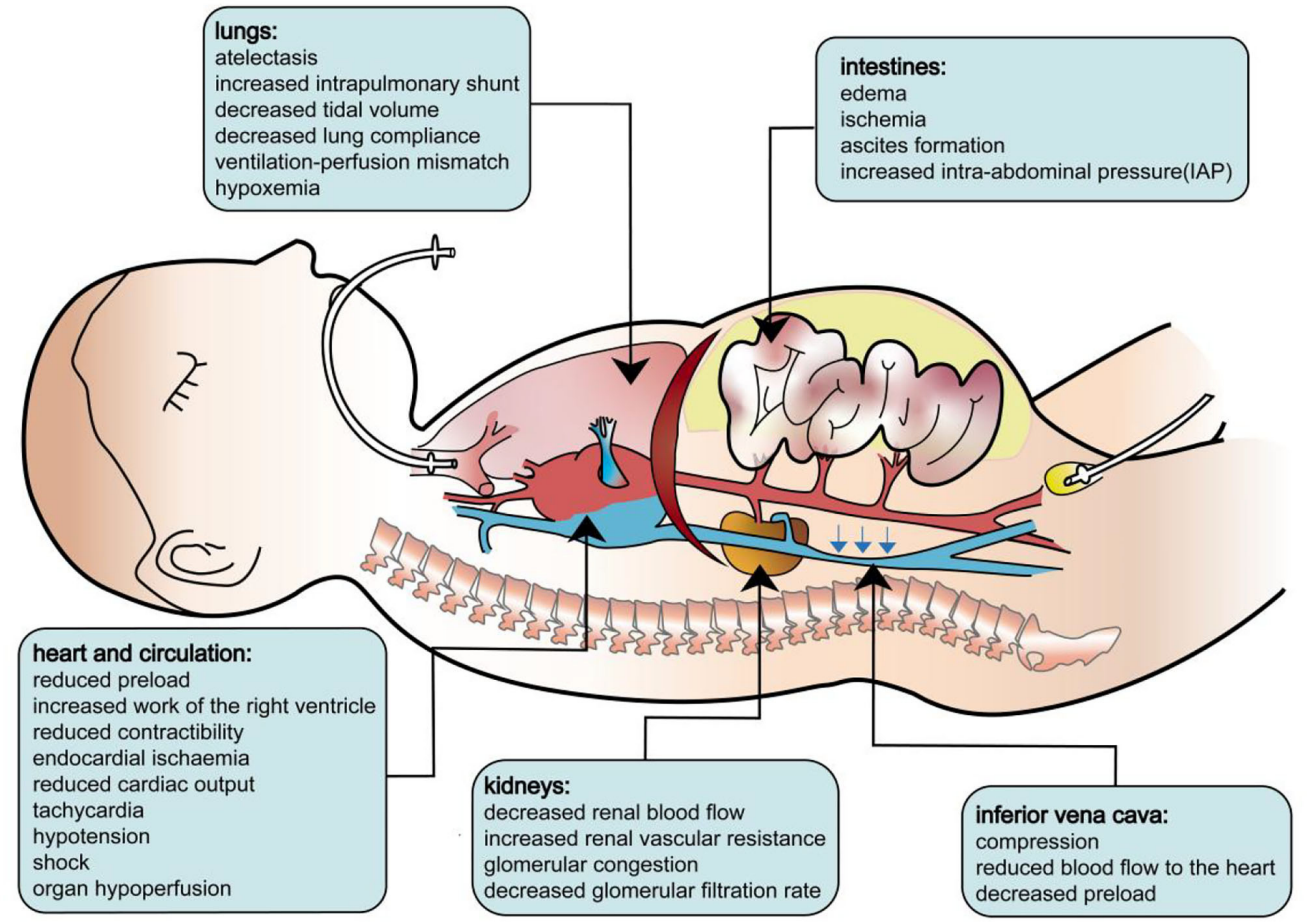
Pathophysiology of abdominal compartment syndrome.

The pathophysiology of secondary ACS is identical to that of primary ACS. As the IAP increases, cardiac output is reduced as a result of decreased central venous return and a consequently diminished right ventricular end-diastolic volume. Initially, high IAP increases systemic vascular resistance, and a “normal” blood pressure may be observed. Paradoxically, intracardiac filling pressures, such as pulmonary artery occlusion pressure and central venous pressure, typically increase with a rising IAP despite the reduced venous return and cardiac output. Then, the increased after load will undermine the contractibility of the cardiac muscle, tampering with the cardiac output. In clinical settings, this will manifest as tachycardia and shock. Beyond the heart, ACS also affects the lungs, kidneys, and other organs. During ACS, the diaphragm shifts cranially, leading to lower respiratory compliance, which increases the effort needed for breathing and the mismatch of perfusion and ventilation. Patients will present with elevated peak pressures, a decreased P/F ratio, hypoxemia, hypercarbia, and atelectasis. IAH may significantly compress the kidney and diminish renal perfusion. Studies have shown that acute renal dysfunction may develop even at relatively low levels of IAP. Renal dysfunction presents as oliguria, progressing to anuria due to a reduced glomerular filtration rate. In addition, ACS also leads to mesenteric, gastrointestinal, and neurological complications secondary to decreased cardiac output and direct compression from IAH (7–10). The pathophysiology of ACS is illustrated in Figure 3.

**Figure 4:**
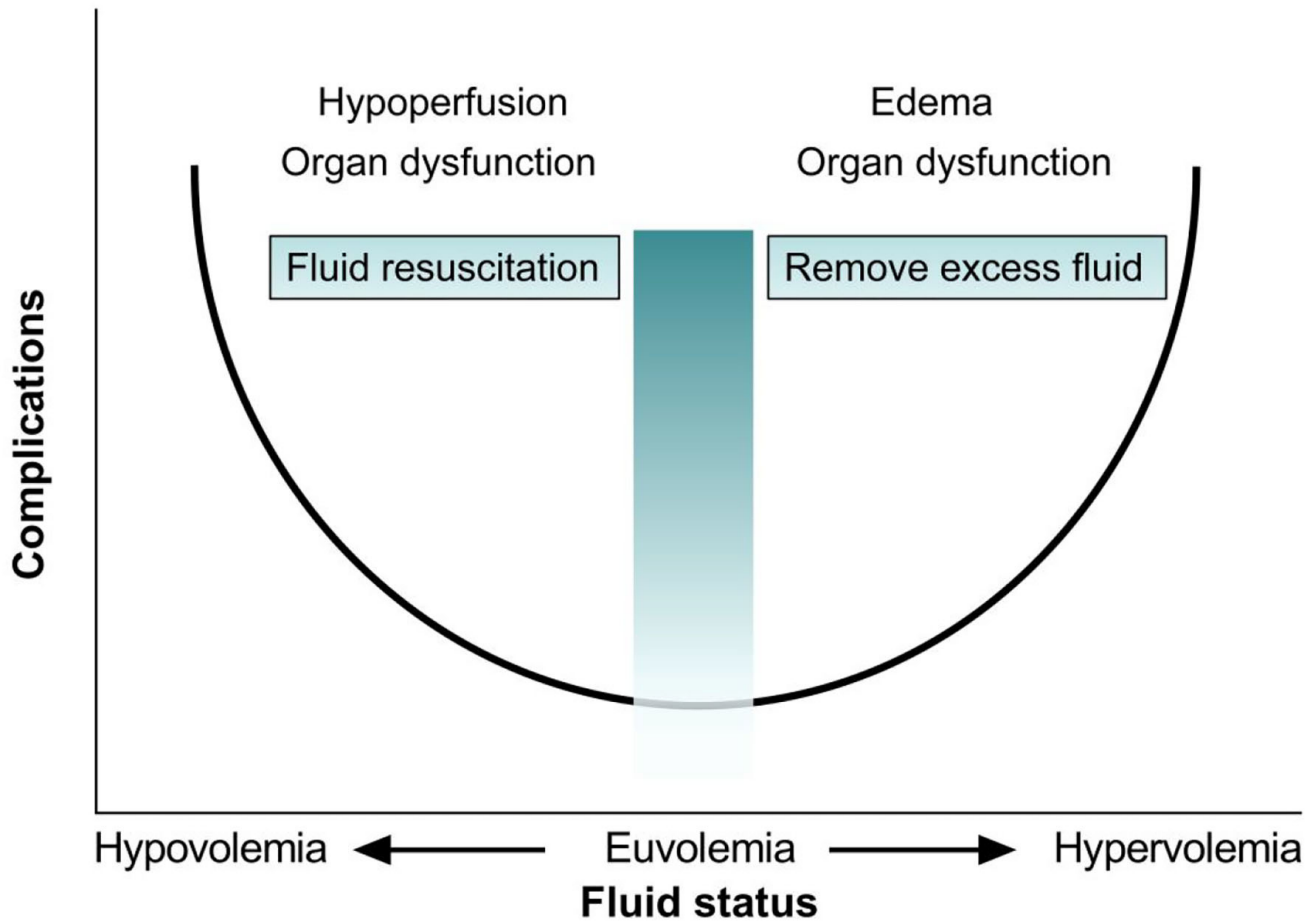
Relation between fluid volume status and complications.

The mechanism of secondary ACS can be related to visceral, peritoneal, and retroperitoneal edema induced by inflammation or fluid resuscitation (11), which are commonly reported in burned children (12). Burn injury is characterized by a hypermetabolic response with physiological, catabolic, and immune effects. Burn areas larger than 15% of the total body surface will lead to a systemic inflammatory response, resulting in disruption of the endothelial glycocalyx, as well as alterations in the structure and function of the extracellular matrix (13–15). This will increase vascular permeability and promote the leakage of plasma fluid to the extracellular space and the interstitial compartment (16). Delayed or insufficient fluid resuscitation may lead to burn shock, poor tissue perfusion, multiple organ dysfunction, and death (17); moreover, fluid overload also results in edema associated with organ dysfunction (as depicted in Figure 4). However, commonly applied fluid resuscitation strategies may be complicated by swelling of the viscera due to inflammation and resuscitation *per se*. Excessive fluid resuscitation, typically using too much crystalloid, may lead to ACS and pulmonary edema (18). This is known as the concept of “fluid creep,” (19) which is reported in 30–90% of severely burned patients (20). Therefore, aggressive fluid resuscitation must be balanced against the possibility of “fluid creep”-induced secondary ACS. The life-threatening ACS in this patient could have resulted from delayed fluid resuscitation, severe burns, and fluid overload.

ACS in burn patients usually develops with three particular events: ~4 days post-injury, following a surgical procedure, and during a period of sepsis. In our case, the child developed ACS 5 days following the burn injury. It is important to know that a reduced urine output or a raised serum lactate may not be because of hypovolemia, IAH could also be a potential cause. Early detection of IAH allows physicians to intervene before the development of ACS. A routine fluid responsiveness assessment [such as passive leg-raising technique (21) and measurement of the inferior vena cava diameters by ultrasound (22)] is helpful for detecting hypovolemia. However, this would become complex and challenging in the advent of ACS. In patients with persistent oliguria and negative fluid responsiveness, IAP should be measured, especially in the aforementioned three circumstances. Current methods of IAP measurement and their reliability have been comprehensively reviewed (1). In brief, bladder pressure (at the end of expiration) is recommended for patients who require ongoing monitoring. In addition, ultrasound may be a useful tool for identifying compression and evaluating bowel movement and abdominal and bowel contents, but its reliability and feasibility in the diagnosis of ACS require further investigation.

Once the diagnosis of ACS is established, medical management should focus on three key areas: management of intraluminal contents, management of the abdominal wall, and management of systemic fluid balance. A comprehensive review of these medical management strategies has been published previously (1). When medical management fails, further advanced management should be considered (1, 23). In general, emergency decompressive surgery is considered but has high morbidity and mortality (24). Some evidence has shown the effectiveness of percutaneous drainage in burned patients with ACS, and this procedure is supported by the World Society of Abdominal Compartment Syndrome (1). In our case, ultrasound-guided percutaneous catheter drainage was successfully performed, with a significant decrease in IAP from 15 to 11 mmHg. However, the clinical condition of the patient did not improve.

CRRT is commonly used for critically ill patients with acute renal failure, fluid overload, and sepsis. In adult burned patients, the effectiveness of CRRT has been reported in reversing septic shock and improving acute renal failure (25). However, its effect on pediatric burned patients is unknown. To the best of our knowledge, this is the first case of the use of CRRT in a pediatric burned patient with ACS. In our case, the patient was prescribed a dose of 20–25 ml/kg·h^−1^ ultrafiltration for 40 h. The excessive fluid was successfully removed, accompanied by a decrease in B-type natriuretic peptide levels, and ACS was reversed. Our case showed that, in pediatric burned patients, CRRT can be beneficial by effectively removing inflammatory mediators, excessive fluid, and accumulated metabolic products while minimizing the effect on hemodynamics. Compared to decompressive laparotomy, CRRT provides a less invasive and promising measure for secondary ACS in severely burned children.

## Conclusion

In severely burned children, secondary ACS can develop after a few days of fluid resuscitation, which requires routine IAP monitoring in these patients. In patients with refractory ACS, CRRT could be considered when other medical treatments fail. This report highlights the role of the interprofessional team in managing severely burned patients.

## Data Availability Statement

The original contributions presented in the study are included in the article/Supplementary Material, further inquiries can be directed to the corresponding authors.

## Ethics Statement

The studies involving human participants were reviewed and approved by the Medical Ethics Research Committee of Huaqiao University Affiliated Strait Hospital. Written informed consent to participate in this study was provided by the participants' legal guardian/next of kin. Written informed consent was obtained from the minor(s)' legal guardian/next of kin for the publication of any potentially identifiable images or data included in this article.

## Author Contributions

SD and CH designed the research. JS, RL, and SH performed the research. JZ, ZP, RS, JC, and YA collected clinical data. JS, XZ, and HL analyzed the data. JS was responsible for patient treatment and drafted the manuscript. CZ and JH reviewed and revised the manuscript. All authors read and approved the final manuscript.

## Funding

This research was supported by Science and Technology Program of Quanzhou (Grant Nos. 2021CT0010, 2019C080R, and 2021N003S).

## Conflict of Interest

The authors declare that the research was conducted in the absence of any commercial or financial relationships that could be construed as a potential conflict of interest.

## Publisher's Note

All claims expressed in this article are solely those of the authors and do not necessarily represent those of their affiliated organizations, or those of the publisher, the editors and the reviewers. Any product that may be evaluated in this article, or claim that may be made by its manufacturer, is not guaranteed or endorsed by the publisher.
