# Supplementary figures and images for: Case Report: Tachycardia, Hypoxemia and Shock in a Severely Burned Pediatric Patient
Source: Front Cardiovasc Med. 2022 Jun 16;9:904400. doi: 10.3389/fcvm.2022.904400 (PMC9243508; doi:10.3389/fcvm.2022.904400)

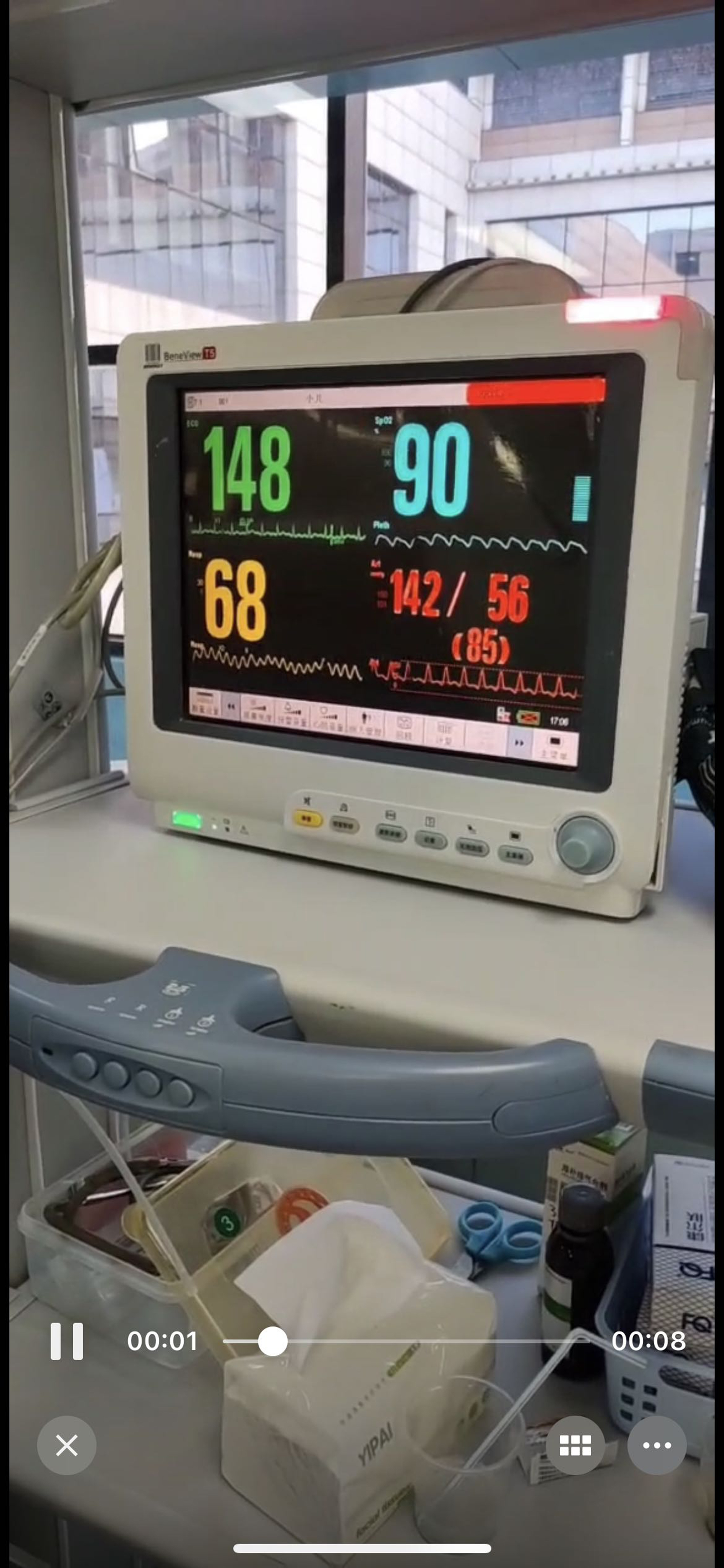

Supplement: Supplementary file 1 [file Image_1.JPEG]

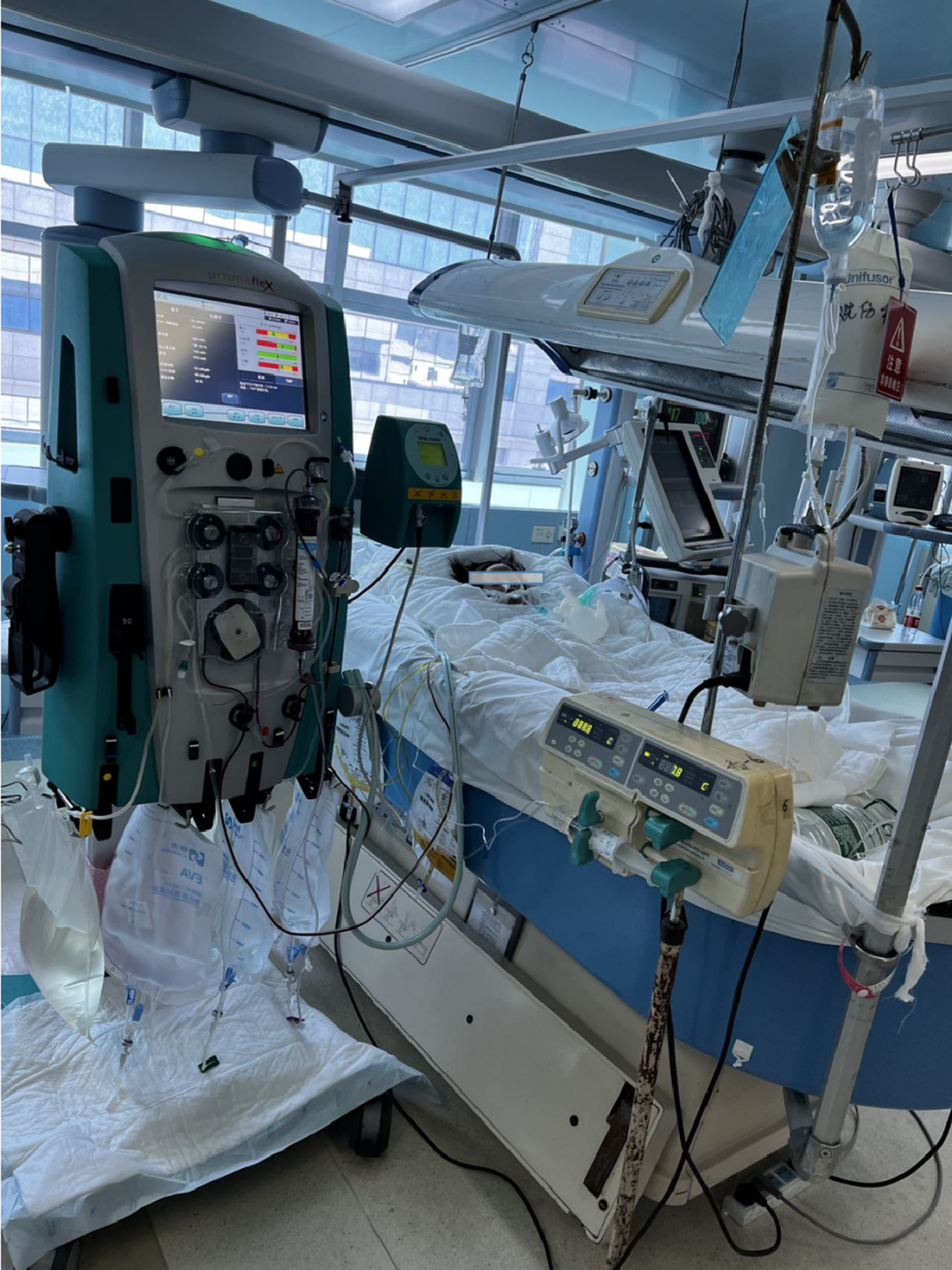

Supplement: Supplementary file 2 [file Image_2.PNG]
